# Chronic salmon calcitonin exerts an antidepressant effect *via* modulating the p38 MAPK signaling pathway

**DOI:** 10.3389/fnmol.2023.1071327

**Published:** 2023-03-10

**Authors:** Wenhui Zhu, Weifen Li, Jian Jiang, Dilong Wang, Xinliang Mao, Jin Zhang, Xunzhi Zhang, Jinlong Chang, Peijia Yao, Xiuyan Yang, Clive Da Costa, Ying Zhang, Jiezhong Yu, Huiliang Li, Shupeng Li, Xinjin Chi, Ningning Li

**Affiliations:** ^1^Tomas Lindahl Nobel Laureate Laboratory, The Seventh Affiliated Hospital, Sun Yat-sen University, Shenzhen, China; ^2^State Key Laboratory of Oncogenomics, School of Chemical Biology and Biotechnology, Peking University Shenzhen Graduate School, Shenzhen, China; ^3^Perfect Life and Health Institute, Zhongshan, Guangdong, China; ^4^College of Life Sciences and Oceanography, Shenzhen University, Shenzhen, China; ^5^The Francis Crick Institute, London, United Kingdom; ^6^The Fourth People’s Hospital of Datong City, Datong, China; ^7^Wolfson Institute for Biomedical Research, Division of Medicine, Faculty of Medical Sciences, University College London, London, United Kingdom; ^8^China-UK Institute for Frontier Science, Shenzhen, China; ^9^Department of Anesthesiology, The Seventh Affiliated Hospital of Sun Yat-sen University, Shenzhen, China; ^10^The Fifth People’s Hospital of Datong City, Datong, China

**Keywords:** depression, salmon calcitonin, p38/JNK signaling pathway, chronic restraint stress, hippocampus

## Abstract

Depression is a common recurrent psychiatric disorder with a high lifetime prevalence and suicide rate. At present, although several traditional clinical drugs such as fluoxetine and ketamine, are widely used, medications with a high efficiency and reduced side effects are of urgent need. Our group has recently reported that a single administration of salmon calcitonin (sCT) could ameliorate a depressive-like phenotype *via* the amylin signaling pathway in a mouse model established by chronic restraint stress (CRS). However, the molecular mechanism underlying the antidepressant effect needs to be addressed. In this study, we investigated the antidepressant potential of sCT applied chronically and its underlying mechanism. In addition, using transcriptomics, we found the MAPK signaling pathway was upregulated in the hippocampus of CRS-treated mice. Further phosphorylation levels of ERK/p38/JNK kinases were also enhanced, and sCT treatment was able only to downregulate the phosphorylation level of p38/JNK, with phosphorylated ERK level unaffected. Finally, we found that the antidepressant effect of sCT was blocked by p38 agonists rather than JNK agonists. These results provide a mechanistic explanation of the antidepressant effect of sCT, suggesting its potential for treating the depressive disorder in the clinic.

## 1. Introduction

Depression is a complex psychiatric disorder characterized by a persistent depressive mood, an abnormal mind, and eating and sleep disturbances, frequently leading to attempted suicide and suicide. Approximately 280 million people worldwide are diagnosed with depression (Evans-Lacko et al., 2017). It is estimated to affect 3.8% of the population around the world, including 5.0% among adults (Evans-Lacko et al., 2017; Hammen, 2018). However, the etiology of depression remains unclear, which raises difficulties for clinical diagnosis and treatment outcomes (Ménard et al., 2016). Moreover, the range of available drugs in the clinic for treatment and therapy is limited, mainly fluoxetine and ketamine. However, they are effective for only 30 to 40% of patients (Emmerzaal et al., 2020). Fluoxetine shows efficacy after 2–4 weeks of treatment, largely by increasing the levels of serotonin (5-HT) and BDNF in the brain, enhancing neuroplasticity and promoting neurogenesis, but has limited beneficial effects in severe patients (Hirschfeld, 2000; Banerjee et al., 2013). Nonetheless, most antidepressants have severe side effects that often outweigh their therapeutic effects, thus hindering prolonged clinical application (Pan et al., 2018). For instance, the induction of nervousness and insomnia by some antidepressants leads to poor toleration in some patients (Papakostas, 2010; Whiskey and Taylor, 2013). Taken together, there is an urgent need to identify more effective and safer therapies.

Calcitonins are a family of peptide hormones produced in vertebrates, including calcitonin, calcitonin gene-related peptide (CGRP), amylin and adrenomedullin. The coding sequences of calcitonin (32 amino acids) and CGRP (37 amino acids) are located in the same gene locus, and two mature peptides are formed by alternative splicing (Amara et al., 1982; Rosenfeld et al., 1983). Calcitonin and CGRP have been widely used in the treatment of neuropsychological diseases as small molecule peptides (Schorscher-Petcu et al., 2009; Hashikawa-Hobara et al., 2015). Animal studies have demonstrated that CGRP treatment reduces immobility time in the forced swim test (FST) in a mouse model of depression, indicating that CGRP has a potential antidepressant effect (Schorscher-Petcu et al., 2009; Hashikawa-Hobara et al., 2015). Additionally, after blocking the CGRP receptor, the antidepressant effect of CGRP disappeared (Hashikawa-Hobara et al., 2015). Clinical studies have shown that calcitonin in the serum of patients with depression is reduced (Mathe et al., 2002). Salmon calcitonin (sCT) has a more robust effect and a longer duration of action than mammalian calcitonin (Christopoulos et al., 1999). At present, sCT is a commonly used drug in the treatment of senile osteoporosis, postmenopausal osteoporosis and hypercalcemia caused by bone metastases (Silva and Becker, 1973; Ellerington et al., 1996). Recent studies have shown that sCT affects alcohol-related behaviors in rodents by modulating dopamine release in the brain regions such as lateral dorsal tegmental area (LDTg), ventral tegmental area (VTA) and nucleus accumbens (NAc) shell (Zakariassen et al., 2020; Kalafateli et al., 2021).

Amylin receptors (AMYRs) consist of calcitonin receptor (CTR) dimerized with receptor activity-modifying proteins (RAMPs; Hay et al., 2018). AMYRs are associated with motivated ingestive behavior and alcohol consumption (Mietlicki-Baase et al., 2017). Small molecule AMYR agonists are considered effective therapeutic candidates, for they can cross the blood–brain barrier and present high specificity (Sonne et al., 2020). In addition, sCT can take effect by activating CTR or/and AMYRs (Arans et al., 2021). Similarly, our group has recently demonstrated that an acute administration of sCT could ameliorate a depressive-like phenotype by activating AMYRs (Jiang et al., 2022). However, the mechanism of the sCT’s antidepressant effect remains to be elucidated.

The MAPK pathway is one of the most critical regulatory pathways in eukaryotic cells. There are at least three distinct MAPK signaling modules, including extracellular regulated protein kinases (ERK), p38 and c-Jun N-terminal kinase (JNK), transducing extracellular signals down to the nucleus where transcription of responsive genes are turned on or off (Oliveira et al., 2008). It has been reported that stress impairs hippocampal function by inducing the expression of MAPK signaling related proteins, leading to activation of apoptosis and neuronal cell death (Abarikwu and Sunny, 2014; Park et al., 2016). Current studies support that inflammatory cytokines and exposure to psychological acute stressors induce the activation of p38/JNK in the brain, and that pro-inflammatory cytokine signaling contributes to the pathogenesis of depression (Chen et al., 2021). As downstream effectors of lipopolysaccharide (LPS) stimulation, phosphorylation levels of JNK and p38 in the hippocampus were increased in mice subjected to chronic unpredictable mild stress (Fu et al., 2013; Naeem et al., 2021). Furthermore, the phosphorylated ERK can shuttle to the nucleus and initiate a series of transcriptional programs, resulting in neuronal damage, which in turn leads to depressive symptoms (Lai et al., 2017; Moniruzzaman et al., 2018).

In the present study, we evaluated the antidepressant potential of chronic administration of sCT, as well as its antidepressant mechanism. Firstly, we showed that chronic sCT treatment could alleviate depressive-like behaviors in CRS-treated mice. Secondly, through transcriptomics and Western blotting analysis, we showed that chronic sCT administration might exert antidepressant effects *via* inhibiting the JNK/P38 signaling pathway. Finally, *via* using p38 and JNK agonists, we confirmed that p38, but not JNK, could be the branch of MAPK pathway responsible for the antidepressant effects of sCT.

## 2. Materials and methods

### 2.1. Animals

Mice were housed in a pathogen-free SPFII animal facility in a condition-controlled room (23 ± 1°C, 50 ± 10% humidity) at the Laboratory Animal Center of Southern University of Science and Technology (SUSTech), Shenzhen, China. A 12 h light/dark cycle was automatically imposed. Mice were maintained in a group of 6 in each ventilated cage and given access to food and water *ad libitum*. All animal experiments were conducted according to the protocols approved by the Animal Care Committee at SUSTech. The Animal Research: Reporting of *In Vivo* Experiments (ARRIVE) guidelines were followed when animal experimentation was designed and performed. Male C57BL/6 J mice were imported from Guangdong Laboratory Animal Center, China.

### 2.2. Chronic restraint stress model

After being acclimated to the facility for a week, mice were subject to the procedures of CRS as previously described (Jiang et al., 2022). In brief, mice were restrained in flexible cylindrical plastic tubes which were fitted to allow them to breathe. Going through the paradigm of CRS, mice were restrained in the tube for 2 h each day and for 14 consecutive days in total. Mice stayed in their own home cages except during the period of CRS.

### 2.3. Open-field test

Open-field test (OFT) was used to measure voluntary movement (Choleris et al., 2001). Distance traveled was recorded for 10 min by EthoVision XT software (Noldus Information Technology, Leesburg, VA, United States).

### 2.4. Forced swimming test

The forced swimming test (FST) was performed according to the reported protocols (Lee et al., 2021). A cylindrical container of transparent plexiglass was used at a diameter of 11.5 cm and a height of 30 cm. A mouse was placed in a container filled with water (water temperature: 22–24°C, height: 20 cm). Their behavior was recorded for 6 min, while the immobility time in the last 5 min was counted and analyzed by the EthoVision XT software.

### 2.5. Tail suspension test

Tail suspension test (TST) was performed according to the reported protocols (Zhang et al., 2020; Li et al., 2021). After habituation, the mouse tail was taped onto the iron hook. The immobility time was recorded for 6 min with EthoVision XT software upon the tail suspension.

### 2.6. Sucrose preference test

In sucrose preference test (SPT), mice were housed individually and habituated to the drinking paradigm in which two water bottles were kept for 24 h, and the bottle position was randomly changed. After the habituation, mice were deprived of water for 24 h, and then two bottles containing water and 1% sucrose, respectively, were placed on the grid of the home cage. Mice were allowed to drink freely for 2 h, and the bottle position was changed during this period. Consumption of water and sucrose solution was measured by weighing the bottles. Sucrose preference was calculated by using the following equation: [sucrose solution intake (g)/(sucrose solution intake (g) + water intake (g))] × 100 (Walker et al., 2019).

### 2.7. Drug administration

sCT (Tocris Bioscience, Bristol, United Kingdom) was diluted in sterile 0.9% saline and injected subcutaneously (*s*.*c*.) at 50 IU/kg bodyweight for 10 consecutive days before completing all behavioral tests. Drug dosage and timing of administration were determined based on previous studies (Kwatra et al., 2020; Jiang et al., 2022). A JNK agonist, anisomycin (AN, MedChemExpress, United States), and a p38 agonist, phorbol 12-myristate 13-acetate (PMA, MedChemExpress, United States) were dissolved in sterile 0.9% saline and injected intraperitoneally (*i*.*p*.) at 0.1 mg/kg, 0.2 mg/kg bodyweight, respectively, for 15 consecutive days before completing the behavioral tests (Guo et al., 2016; Chen et al., 2022).

### 2.8. Western blotting assay

Total protein was isolated from the dissected tissues using moderate-intensity RIPA buffer (Beyotime, Shanghai, China) containing protease inhibitor and phosphatase inhibitor (MedChemExpress, New Jersey, United States). After being centrifuged at 12,000 rpm at 4°C for 15 min, supernatants were collected, and protein concentration was determined using Pierce™ BCA Protein Assay Kit (Thermo Fisher Scientific, MA, United States). Proteins were separated by sodium dodecyl sulfate–polyacrylamide gel electrophoresis (SDS-PAGE), transferred to a polyvinylidene fluoride (PVDF) membrane (Merck Millipore, Guangzhou, China), and blocked in 5% fat-free milk in 1× PBST (0.1% Tween20 in phosphate buffer solution) for 1 h at room temperature. The blots were incubated with primary antibodies for p-p38 (Abcam, Cambridge, United Kingdom) and ERK1/2, p-ERK1/2, JNK, p-JNK, PTEN, p-PTEN, PI3K, p-PI3K, AKT, p-AKT, mTOR, p-mTOR, BDNF, PSD95, snap25 and synapsin-1 (Cell Signaling Technology, Massachusetts, United States) in a 5% bovine serum albumin (BSA) solution overnight at 4°C. On the second day, the blots were washed with 1× PBST for 3 times and 10 min each, and incubated with HRP-conjugated secondary antibodies for 1 h (anti-rabbit IgG, ProteinTech Group, Inc., Wuhan, China). Immunodetection was performed using a super ECL detection reagent (Yeasen Biotech, Shanghai, China), and the signal was detected with a ChemiDoc™ Touch Imaging System (Bio-Rad, Shanghai, China).

### 2.9. mRNA sequencing

The hippocampal tissues from the CRS group and the control group were excised. Library preparation and transcriptome sequencing were performed at the Shanghai Applied Protein Technology Co., Ltd (APTBIO, Shanghai, China). Total RNA was extracted from hippocampus using TRIzol reagent (Thermo Fisher Scientific, United States). To construct the RNA-seq library, a total amount of 2 μg RNA per sample was used as input material for high-throughput sequencing with the HiSeq 2000 sequencing system (Illumina, Shanghai) according to the manufacturer’s recommendations. Then, the libraries were then quantified and pooled. Paired-end sequencing of the library was performed on the HiSeq XTen sequencers (Illumina, San Diego, CA). Raw data were processed to filter out reads with low quality and clean data were aligned to the mouse genome using HISAT2, and reads numbers mapped to each gene were counted by FeatureCounts. DESeq2 was used to determine differentially expressed genes (DEGs) between two groups. Genes were considered as significantly differentially expressed if *p* value <0.05 and | log2FC (fold change) | > 0.583. The ggplot2 package and pheatmap package were used to create a volcano plot and a heatmap, respectively. Kyoto Encyclopedia of Gene and Genomes (KEGG, kegg.jp) pathway analysis were performed on the free online website^1^ (Xu et al., 2019; Wang et al., 2021; Zheng et al., 2021).

### 2.10. Statistics

The data from the behavioral tests and Western blotting, among 3 or 4 groups, were analyzed with one-way ANOVA followed by Tukey’s multiple comparison tests. Behavioral tests between the control + saline and control + sCT groups were analyzed by unpaired t test. Statistical analysis was performed using GraphPad Prism 8 (GraphPad Software, La Jolla, CA, United States), and *p* < 0.05 was considered statistically significant. All data are represented as the mean ± SEM.

## 3. Results

### 3.1. The locomotor activity was not altered by chronic salmon calcitonin treatment in the chronic restraint stress-treated mice

**Figure 1:**
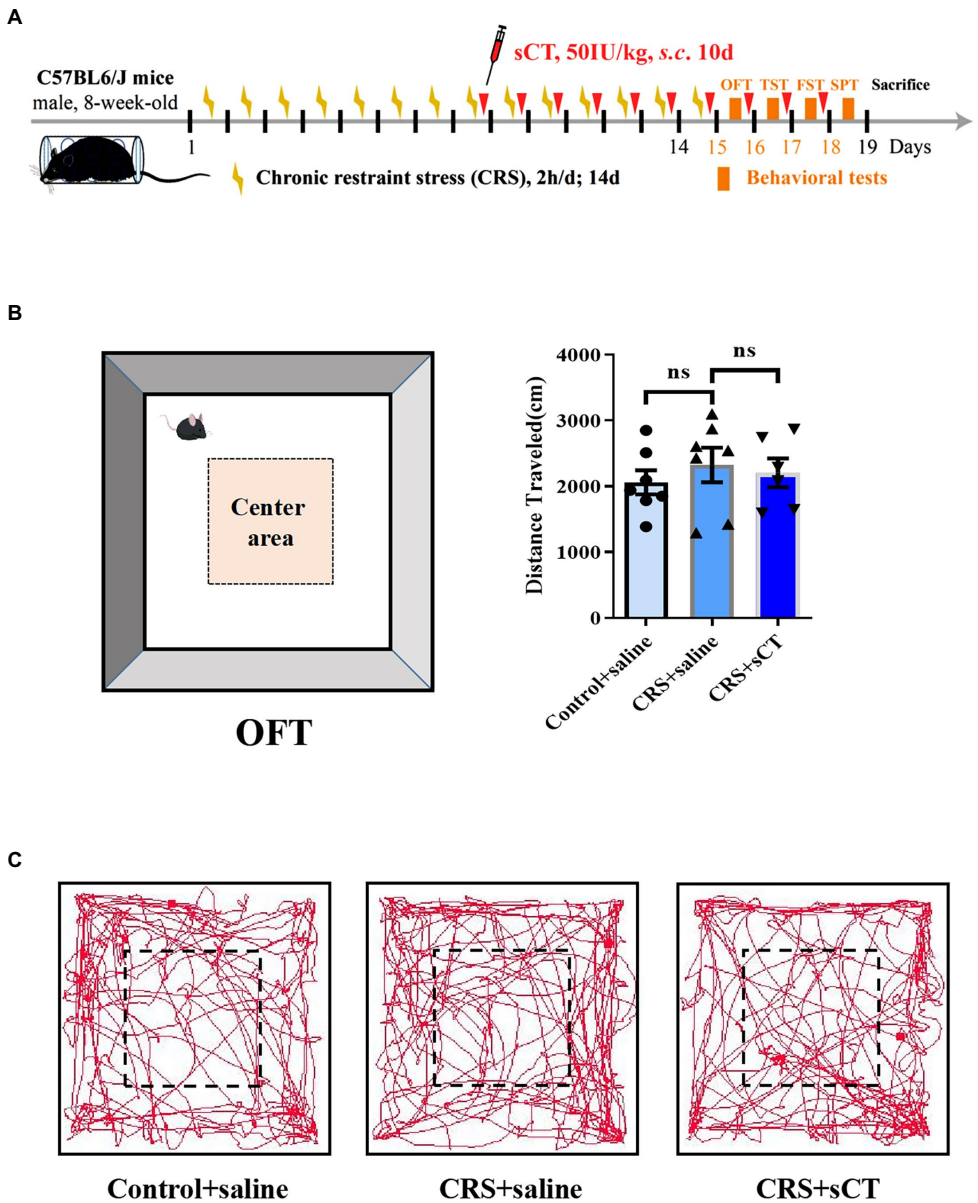
Locomotion level did not change in CRS-treated mice. **(A)** Schematic diagram showing experimental procedures of the CRS-treated mice model. **(B)** The distance traveled in OFT did not change in CRS-treated mice (Control + saline: *n* = 7; Depression + saline: *n* = 7; Depression + sCT: *n* = 6). **(C)** Representative images of movement trace in OFT. The data were analyzed by one-way ANOVA with Tukey’s multiple comparison tests. ns, no significance.

To illustrate the therapeutic effect of chronic sCT on depression, an animal model of CRS was established. Male mice were subject to CRS for 2 weeks. In addition, sCT was subcutaneously (*s*.*c*.) injected at 50 IU/kg bodyweight for 10 consecutive days (Figure 1A). To assess and compare any possible changes in the locomotor activity, we performed OFT in the CRS-treated mice relative to the control group. As expected, no significant changes were found in the total distance traveled between the CRS group and the control group. Further, chronic sCT administration did not affect the locomotion level in the CRS-treated mice (*F*_(2,17)_ = 0.3662, *p* > 0.05; Figures 1B,C).

### 3.2. Chronic salmon calcitonin treatment exerts an antidepressant effect on chronic restraint stress-treated mice but dose not affect non-treatment control mice

**Figure 2:**
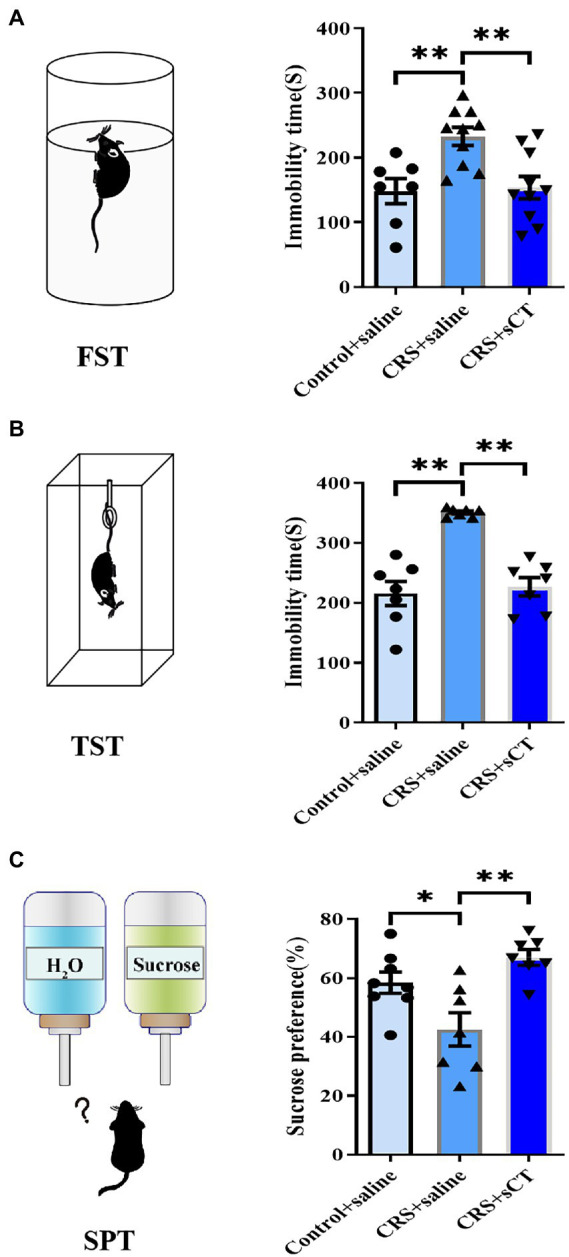
Chronic sCT treatment had an antidepressant effect in CRS-treated mice. **(A)** In FST, the immobility time of CRS-treated mice was increased and was decreased after sCT treatment (Control + saline: *n* = 7; Depression + saline: *n* = 10; Depression + sCT: *n* = 10). **(B)** In TST, the immobility time of CRS-treated mice was also significantly increased and was decreased after sCT treatment (Control + saline: *n* = 7; Depression + saline: *n* = 7; Depression + sCT: *n* = 7). **(C)** In SPT, the sucrose preference of CRS-treated mice was decreased significantly and was increased after sCT treatment (Control + saline: *n* = 8; Depression + saline: *n* = 7; Depression + sCT: *n* = 7). The data were analyzed by one-way ANOVA with Tukey’s multiple comparison tests. *p < 0.05, **p < 0.01.

Learned helplessness and anhedonia are two main core symptoms of depressive disorder. The FST/TST and SPT are common paradigms to evaluate the behavioral phenotype of learned helplessness and anhedonia, respectively (Martins and Brijesh, 2018). Hence, we used TST, FST and SPT to assess the antidepressant effect of sCT in CRS-treated mice vs. control group. In the FST and TST, immobility time in CRS-treated mice was significantly reduced after sCT treatment (*F*_(2, 24)_ = 8.290, *p* < 0.01; *F*_(2, 18)_ = 25.98, *p* < 0.0001; Figures 2A,B). In the SPT, the sucrose preference ratio was significantly lowered in CRS-treated mice, suggesting the antidepressant effect in mice (*F*_(2, 19)_ = 8.677, *p* < 0.01; Figure 2C).

Notably, sCT treatment did not change the distance traveled by control mice compared to that of saline treatment group (*t*_(11)_ = 0.607, *p >* 0.05, Supplementary Figure S1A). In the FST and TST, the immobility time was not changed after sCT treatment compared with the control group (*t*_(11)_ = 0.6073, *p >* 0.05; *t*_(12)_ = 1.049, *p >* 0.05, Supplementary Figures S1B,C). In the SPT, the sucrose preference of control mice was not changed after sCT treatment (*t*_(14)_ = 0.056, *p >* 0.05, Supplementary Figure 1D).

### 3.3. The PI3K/Akt and MAPK signaling pathway were significantly changed in chronic restraint stress-treated mice

**Figure 3:**
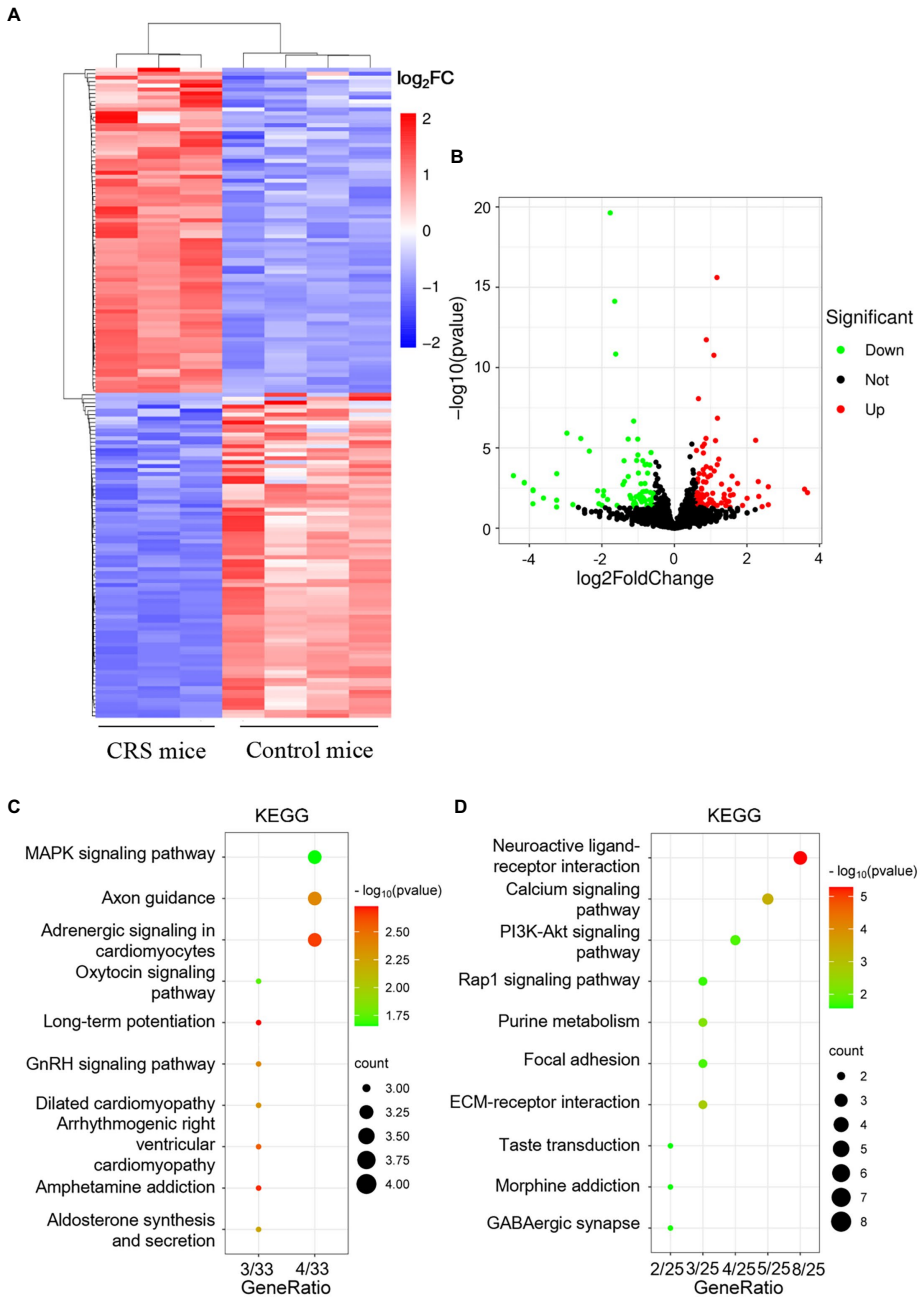
mRNA expression profile analysis of the hippocampus in control mice and CRS-treated mice. **(A)** The heatmap of DEGs between Control mice and CRS-treated mice. **(B)** The volcano map of genes between Control mice and CRS-treated mice. **(C,D)** The KEGG enrichment analysis of up-regulated genes **(C)** and down-regulated genes **(D)**.

To elucidate the molecular mechanism underlying the effect of sCT on the CRS-treated mice, we first sought to identify significantly changed signaling pathways between CRS-treated mice and control mice. Considering that the hippocampus is inextricably involved in the pathogenesis of depression (Spalding et al., 2013; Liu et al., 2017), we performed RNA sequencing to compare the hippocampal transcriptomic profiling of the two groups of mice. DESeq2 was used to analyze the differentially expressed genes (DEGs). The results of cluster analysis showed that the mRNA profiles of CRS-treated mice were significantly different from those in the control group (Figure 3A). With the screening criteria set at *p* value <0.05 and | log2FC | > 0.583, we further found 164 significant DEGs among which 82 genes were up-regulated, and 82 genes down-regulated in the CRS-treated mice (volcano plot; Figure 3B). KEGG enrichment analysis from 82 up-regulated genes showed that these genes were involved in signaling pathways, including MAPK (scatter plot; Figure 3C). It was reported that activation of MAPK pathway could induce phosphorylation of NF-κB, leading to neuroinflammation and the subsequent development of depression-like behaviors in rodents (Olianas et al., 2019). The ERK/NF-κB pathway was also reported to be activated in the chronic mild stress (CMS) model of depression (Su et al., 2017). Interestingly, KEGG enrichment analysis from 82 down-regulated genes showed that these genes were involved in signaling pathways, including PI3K/Akt (scatter plot; Figure 3D). Similarly, the CRS-treated ICR mice were subjected to cognitive deficits with the decreased AKT/mTOR in hipppocampus (Huang et al., 2022). The analysis found that the PI3K/Akt and MAPK signaling pathway were significantly changed in CRS-treated mice.

### 3.4. Chronic salmon calcitonin treatment selectively blocks the p38/JNK signaling pathway in chronic restraint stress-treated mice

**Figure 4:**
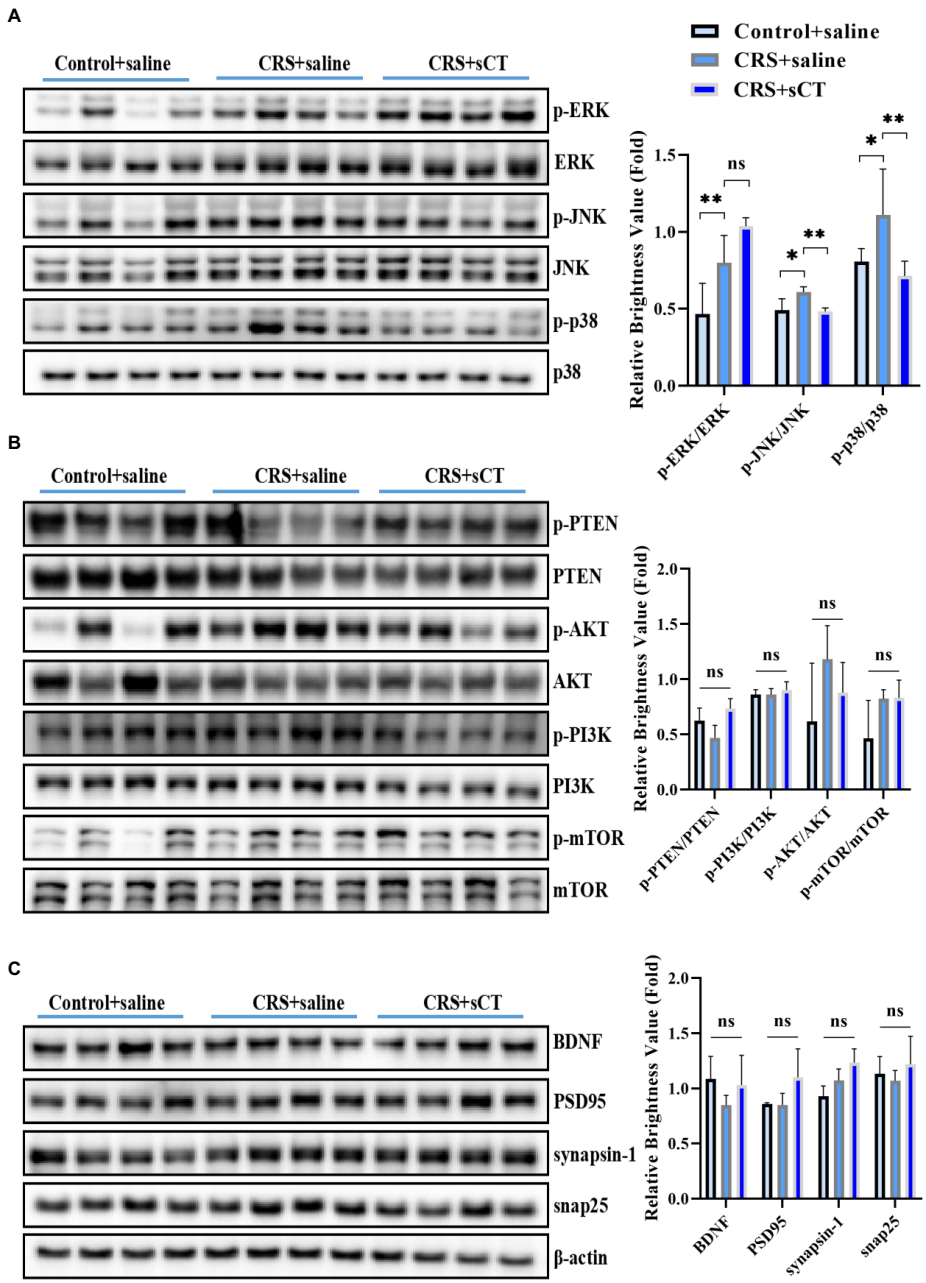
sCT treatment blocked the p38/JNK signaling pathway in the hippocampus of CRS-treated mice. **(A)** The phosphorylation levels of ERK/p38/JNK in the hippocampus of control mice, CRS-treated mice and CRS-treated mice with sCT treatment. **(B)** The phosphorylation levels of PTEN/PI3K/AKT/mTOR in the hippocampus of control mice, CRS-treated mice, and CRS-treated mice with sCT treatment. **(C)** The expression of BDNF/PSD95/synapsin-1/snap25 in the hippocampus of control mice, CRS-treated mice and CRS-treated mice with sCT treatment. The data were analyzed by one-way ANOVA with Tukey’s multiple comparisons tests. *p < 0.05, **p < 0.01.

Considering our finding that the ERK1/2 signaling pathway analysis was significantly up-regulated in the CRS-treated mice, as well as literature that activation of the ERK/p38/JNK pathway was involved in the neuroinflammatory regulation of depression (Liu et al., 2018), we focused on ERK/p38/JNK pathway in this study. Western blotting analysis showed that compared with control mice, the ratios of p-ERK/ERK, p-JNK/JNK and p-p38/p38 were significantly increased in CRS-treated mice (*F*_(2,8)_ = 12.13, *p <* 0.05; *F*_(2,8)_ = 9.699, *p <* 0.05; *F*_(2,9)_ = 4.602, *p <* 0.05; Figure 4A), suggesting an overall induction in the phosphorylation level of the MAPK signaling pathway. However, the ratio of p-PTEN/PTEN, p-PI3K/PI3K, p-AKT/AKT, p-mTOR/mTOR were not significantly changed between CRS-treated mice and control mice (*F*_(2,9)_ = 0.7067, *p >* 0.05; *F*_(2,9)_ = 0.6413, *p >* 0.05; *F*_(2,9)_ = 2.146, *p >* 0.05; *F*_(2,9)_ = 3.488, *p >* 0.05; Figure 4B). In addition, the expression of BDNF, PSD95, synapsin-1, snap25 were not changed (*F*_(2,9)_ = 1.483, *p >* 0.05; *F*_(2,9)_ = 3.093, *p >* 0.05; *F*_(2,9)_ = 2.222, *p >* 0.05; *F*_(2,9)_ = 0.7261, *p >* 0.05; Figure 4C). Strikingly, after sCT treatment, the phosphorylation levels of JNK and p38 in the hippocampus of CRS-treated mice were significantly decreased to a level near the control group (*F*_(2,8)_ = 9.699, *p <* 0.05; *F*_(2,9)_ = 4.602, *p <* 0.05; Figure 4A), whilst the phosphorylation of ERK1/2, PTEN, PI3K, AKT, mTOR, and the expression of BDNF, PSD95, synapsin-1, snap25 were remained unchanged (Figures 4A–C). Taken together, our results indicated that chronic sCT primarily acted on the p38/JNK signaling pathway in CRS-treated mice.

### 3.5. Chronic salmon calcitonin exerts antidepressant effects in the chronic restraint stress model by decreasing phospho-p38

**Figure 5:**
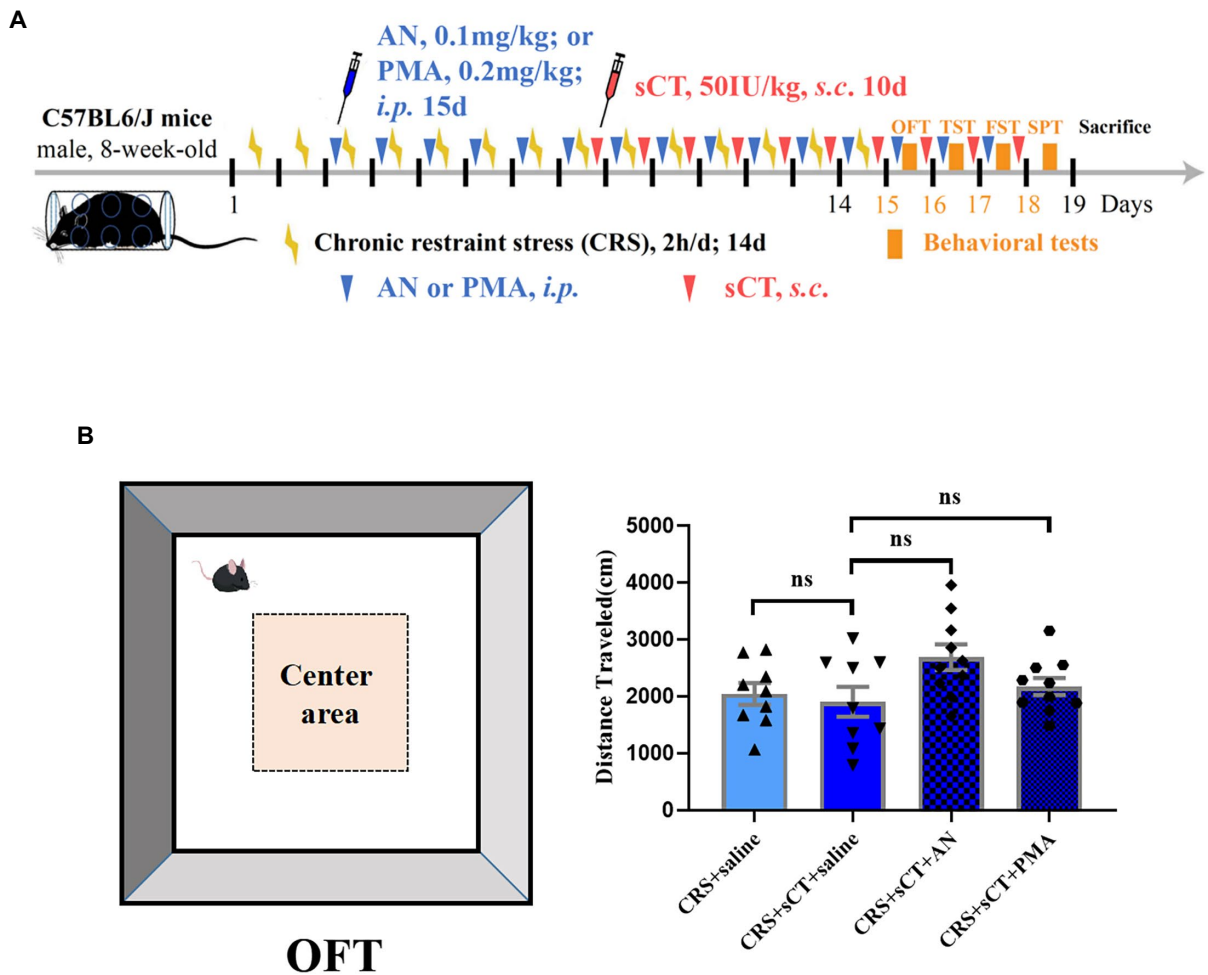
Locomotion level did not change in CRS-treated mice after AN or PMA treatment. **(A)** Timeline of CRS exposure, sCT, AN, PMA administration, and behavioral tests. **(B)** The distance traveled in OFT did not change in CRS-treated mice after AN or PMA treatment (Depression + saline: *n* = 9; Depression + sCT + saline: *n* = 9; Depression + sCT + AN: n = 10; Depression + sCT + PMA: *n* = 10). The data were analyzed by one-way ANOVA with Tukey’s multiple comparison tests. ns, no significance.

**Figure 6:**
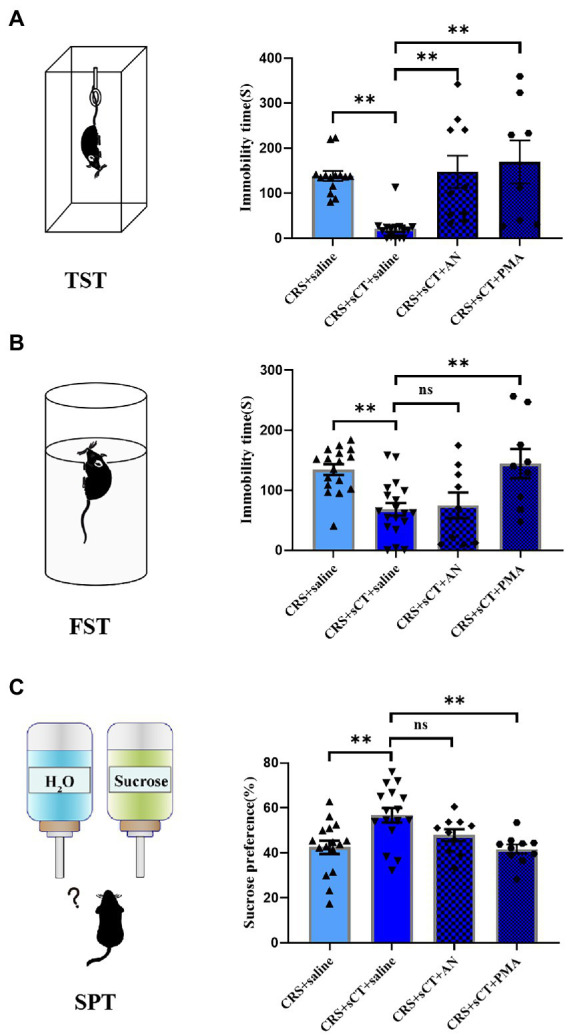
PMA treatment could block the antidepressant effect of sCT in CRS-treated mice. **(A)** In TST, the immobility time of CRS-treated mice with sCT treatment was increased after PMA or AN treatment (Depression + saline: *n* = 14; Depression + sCT + saline: *n* = 15; Depression + sCT + AN: *n* = 10; Depression + sCT + PMA: *n* = 8). **(B)** In FST, the immobility time of CRS-treated mice with sCT treatment was increased after PMA treatment (Depression + saline: *n* = 17; Depression + sCT + saline: *n* = 19; Depression + sCT + AN: n = 9; Depression + sCT + PMA: *n* = 9). **(C)** In SPT, the surose preference of CRS-treated mice with sCT treatment was decreased significantly after PMA treatment (Depression + saline: *n* = 16; Depression + sCT + saline: *n* = 16; Depression + sCT + AN: *n* = 10; Depression + sCT + PMA: *n* = 10). The data were analyzed by one-way ANOVA with Tukey’s multiple comparison tests. ns, no significance, *p < 0.05, **p < 0.01.

To investigate whether the antidepressant effect of chronic sCT is dependent on the JNK/p38 signaling pathway *in vivo*, a JNK agonist, AN, a p38 agonist, PMA or saline, as a negative control was injected intraperitoneally for 15 consecutive days prior to sCT treatment as described above (Figure 5A). We found that AN or PMA did not affect the locomoter activity of CRS-treated mice with sCT treatment (*F*_(3, 34)_ = 2.703, *p >* 0.05; Figure 5B). However, the TST results showed that the immobile time of mice was significantly prolonged upon AN or PMA perturbation, compared to that of the saline treatment group, indicating that the anti-depressant effect of sCT on CRS-treated mice could be abolished by AN or PMA (*F*_(3, 43)_ = 8.960, PMA, *p <* 0.01, AN, *p <* 0.01; Figure 6A). Intriguingly, the results of FST and SPT showed that PMA, but not AN, effectively blocked the antidepressant function of sCT (*F*_(3, 50)_ = 7.661, PMA, *p <* 0.01, AN, *p >* 0.05; Figure 6B; *F*_(3, 48)_ = 5.974, PMA, *p <* 0.01, AN, *p >* 0.05; Figure 6C). Collectively, these results delineated that sCT may exert its antidepressant effects selectively through the p38 rather than the JNK signaling pathway.

## 4. Discussion

Depression is a common debilitating neuropsychiatric disorder with an increased incidence year on year (Hammen, 2018). Our group has recently demonstrated that a single administration of sCT could ameliorate a depressive-like phenotype probably *via* the amylin signaling pathway (Jiang et al., 2022). The potential role of the calcitonin-AMYR axis in depression has been delineated by exploiting both agonists (e.g., sCT) and inhibitors/antagonists of AMYR (e.g., AC187; Hay et al., 2005; Jiang et al., 2022). However, the mechanism of its long-term action remains largely unclear. In this study, we evaluated the anti-depressant potential of chronic sCT treatment. Strikingly, we found that the immobility time of CRS-treated mice was significantly decreased in the FST and TST, and the sucrose preference rate was significantly increased in SPT after chronic application of sCT, while the locomoter activity in CRS-treated mice was not affected (Figures 1, 2), suggesting the profound antidepressant effects of chronic sCT on the CRS-treated mice. Nonetheless, chronic sCT did not affect the aforementioned behaviors of the control mice (Supplementary Figure S1). Further, we sought for the underlying mechanism on the altered molecular networks between the CRS-treated mice and the control group. Our RNA-seq analysis between these two groups revealed significantly enriched transcriptomic profiles, such as MAPK signaling pathway (Figure 3). To validate our findings, Western blotting analysis showed that the phosphorylation level of ERK/p38/JNK molecules was increased in the hippocampus of CRS-treated mice relative to the control group; however, chronic sCT treatment could only reverse the phosphorylation status of p38/JNK in the hippocampus of CRS-treated mice (Figure 4). Notably, the p38 agonist, PMA, rather than JNK agonist, could potently abrogate the antidepressant effects of sCT (Figures 5, 6).

In recent years, a growing number of studies have found that sCT plays an important role in regulating the physiological functions of the central nervous system (Zakariassen et al., 2020; Kalafateli et al., 2021). Taskiran and Filiz showed that sCT had antiepileptic activity in Pentylenetetrazole (PTZ)-induced epileptic seizures in rats through excitatory-inhibitory, oxidative stress and neuroinflammation pathways. After PTZ induced epileptic seizures, sCT even displayed neuroprotective effects on hippocampal neurons (Taskiran et al., 2020; Filiz and Karabulut, 2022). Moreover, sCT can regulate the alcohol intake of mice by activating the mesolimbic dopamine system (Kalafateli et al., 2021). In this study, we focused on chronic calcitonin treatment that significantly rescued the depressant behaviors of our depression mouse model (Figure 2), reminiscent of the finding of our previous study on the acute calcitonin administration (Jiang et al., 2022). However, sCT did not affect the immobility time of the healthy control mice in TST and FST, as well as sucrose preference rate in SPT (Supplementary Figure S1), suggesting that chronic calcitonin administration could be a safe therapeutic strategy, not generating anti-or pro-depressant behaviors to healthy peers. Further, we found that sCT treatment did not affect the locomoter activities of both CRS-treated and non-treatment mice (Figure 1; Supplementary Figure S1), partially in keeping with existing literature on that sCT treatment for 5 days did not affect locomotor activity in wild-type mice (Kalafateli et al., 2020), and also suggesting that the antidepressant effect of calcitonin is highly likely to be through the central nervous system, but not *via* muscle or bone.

In humans and various animal models of depression, the middle prefrontal cortex and hippocampus are two core regions associated with the pathophysiology and progression of depression (Belleau et al., 2019). Subsequent alterations of signaling pathways often occur here, such as MAPKs, encompassing at least ERK, p38, and JNK branches. Upon activation of upstream kinases, the different subfamilies regulate the expression of multiple pro-inflammatory mediators and apoptotic signals (Wang and Mao, 2019; Falcicchia et al., 2020; Behl et al., 2022). Stress induces the activation of the MAPK signaling pathway, furthering the activation of apoptosis, resulting in neuronal cell death, and thereby impairing hippocampal function (Duman et al., 2007; Villas Boas et al., 2019). In this study, our transcriptomic profiling revealed the enrichment of MAPK signaling pathways (Figure 3) and our Western blotting analysis confirmed that the phosphorylation level of ERK1/2, p38 and JNK was significantly increased in the CRS-treated mice (Figure 4). In keeping, Bravo et al. (2009) showed the augmented level of ERK phosphorylation in the hippocampus of CRS-treated rats. In addition, studies utilizing other common depression paradigms also reported the increased phospho-ERK in the hippocampus of chronic unpredictable mild stress (CUMS) rats (Yuan and Yuan, 2022), chronic water immersion restraint stress (CWIRS) mice (Mao et al., 2020), and even mice acutely exposed to FST and TST (Galeotti and Ghelardini, 2012). Nonetheless, decline in phospho-ERK was observed using a similar restraint stress model (Abe-Higuchi et al., 2016; Cui et al., 2023). This discrepancy could possibly due to the modeling time that was manipulated differently in such restraint stress models. Further, we postulate that the dynamics of phospho-ERK levels might come down to the presence of the multi-subtypes and stages of the major depression disorder clinically. Albeit the outcome of ERK phosphorylation is fickle, hippocampal ERK activation is thought to be inextricably involved in the induction of pro-depressant behavior in rodent models (Bravo et al., 2009; Galeotti and Ghelardini, 2012).

Increasing evidence revealed the indispensable roles of the other two members of the MAPK family, p38 and JNK, in regulation of depression (Ménard et al., 2016). Consistent with our observations, some reports indicated that the phosphorylation levels of JNK and p38 were significantly increased in the hippocampus of rodent models after exposure to restraint stress (Tan et al., 2017; Salehpour et al., 2019), chronic mild stress (Martins and Brijesh, 2018) or swim stress (Shen et al., 2004; Bruchas et al., 2007). Inhibition of the activity of JNK and p38 in wild-type mice could result in antidepressant-like behavior (Galeotti and Ghelardini, 2012), indicative their role in induction of depression. Notably, some therapeutic drugs or compounds may relieve depressive symptoms *via* p38 or JNK pathways. Tan et al. found that ketamine alleviated depressive-like behaviors *via* abrogating the activated p38 signaling pathway in the CRS model (Tan et al., 2017). A few studies reported that fluoxetine could improve depression-like behavior by inhibiting the p38/JNK signaling pathway in the depression-like mice (Moretti et al., 2016; Athira et al., 2018). Hesperidin, as a major dietary bioflavonoid that could cross the blood–brain barrier (Youdim et al., 2003), has been shown to exhibit antidepressant effects on CRS-and LPS-treated mice by inhibiting JNK/p38 signaling pathway (Kwatra et al., 2020, 2021). In keeping, we showed that sCT treatment could block the augmented levels of phospho-p38/JNK in the CRS model, while the phosphorylation levels of ERK1/2 were not changed significantly (Figure 4A), possibly due to the antidepressant effects of chronic sCT largely depending on suppressing the p38 or JNK signaling pathway. Strikingly, PMA, as a p38 agonist that could penetrate the blood–brain barrier (Guo et al., 2016), presented more effective blockade of the antidepressant effect of sCT compared to that of the JNK agonist AN (Chen et al., 2022; Figure 6). Of note, PMA and AN did not affect the locomotor activity of mice (Figure 5).

Furthermore, a plethora of studies showed that the PTEN/AKT/mTOR or BDNF/synaptophysin pathway was not changed in a variety of depression models compared to that of the control group (Ju et al., 2022; Olave et al., 2022; Yang et al., 2022). Likewise, we found that the phosphorylation levels of the PTEN/PI3K/AKT/mTOR pathway (Figure 4B) or the expression levels of BDNF/PSD95/synapsin1/snap25 pathway (Figure 4C) remained unchanged between CRS-treated mice and the non-treatment peers. However, there were reports showing that the AKT or BDNF/PSD95 pathway was inhibited in the CRS-treated mice and other depression models (Cunha et al., 2016; Ludka et al., 2016; Song et al., 2020; Li et al., 2020a,b). Nonetheless, data from this study suggested that these aforementioned pathways were neither involved in CRS-treated mice, nor changed by sCT treatment.

Here, we showed activation of the three branches of MAPK signaling pathway (ERK/p38/JNK) in the CRS mouse model, and demonstrated that chronic sCT treatment alleviated the depression-like behaviors of the mice by mainly inhibiting p38 phosphorylation in the hippocampus. Our findings may pave the way for future studies to determine the detailed mechanisms underlying sCT in regulation of the p38 signals, and shed light on potential targeted therapeutic strategies for depression.

## Data availability statement

The data presented in the study are deposited in the CNGB Sequence Archive (CNSA) of China National GeneBank DataBase (CNGBdb), accession number CNP0003857 (https://db.cngb.org/search/project/CNP0003857).

## Ethics statement

The animal study was reviewed and approved by Laboratory Animal Center of Southern University of Science and Technology (SUSTech), Shenzhen, China.

## Author contributions

JZ, XC, and NL designed the project. WZ, WL, NL, and JJ designed the experiments. WZ, JZ, WL, DW, XY, JJ, JC, PY, XZ, and XM performed the experimental work. WZ, WL, DW, JY, and XM analyzed the results. WZ wrote the first draft of the manuscript. NL, HL, SL, XC, and CD revised the manuscript. All authors contributed to and have approved the final manuscript.

## Funding

This work was supported by grants from the Hundred Talents Program of Sun Yat-sen University (392007, NL), National Natural Science Foundation of China (81874176 and 82072766, NL), Shenzhen Sanming Project of Medicine (SZSM201911003, NL), Shenzhen Science, Technology and Innovation Commission (SZSTI) Basic Research Program (JCYJ20190809154411427, NL; JCYJ2019089143601759, Yunping Fan; JCYJ20210324123208022, Tao Wang), and Basic and Applied Basic Research Fund Committee of Guangdong Province (2020A1515110161, Jun Ju).

## Conflict of interest

The authors declare that the research was conducted in the absence of any commercial or financial relationships that could be construed as a potential conflict of interest.

The reviewer G-PL declared a past co-authorship with the author SL to the handling editor.

## Publisher’s note

All claims expressed in this article are solely those of the authors and do not necessarily represent those of their affiliated organizations, or those of the publisher, the editors and the reviewers. Any product that may be evaluated in this article, or claim that may be made by its manufacturer, is not guaranteed or endorsed by the publisher.

## Footnotes

^1^ www.bioinformatics.com
