# Supplementary material for: Chronic salmon calcitonin exerts an antidepressant effect via modulating the p38 MAPK signaling pathway
Source: Front Mol Neurosci. 2023 Mar 10;16:1071327. doi: 10.3389/fnmol.2023.1071327 (PMC10036804; doi:10.3389/fnmol.2023.1071327)
Supplement: Supplementary file 6 [file Data_Sheet_2.docx]

Supplementary Material

# Supplementary Figures


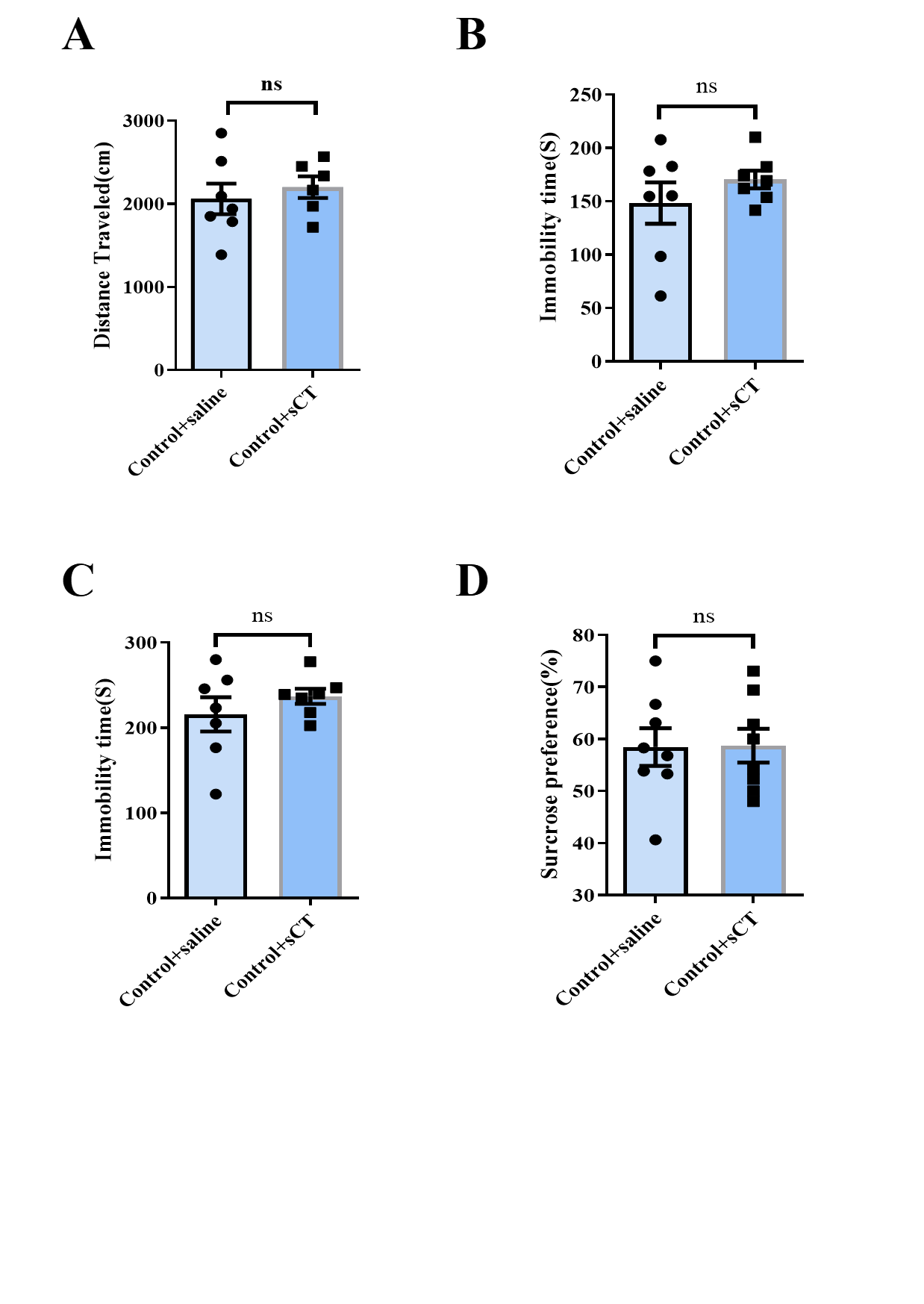


**Figure S1. Chronic sCT does not affect behavioral phenotypes on control mice**

1. The distance travelled in OFT did not change with application of sCT (Control + saline: n = 7; Control + sCT: n = 6). (B) In FST, sCT could not change the immobility time of control mice (Control + saline: n = 7; Control + sCT: n = 7). (C) In TST, sCT also could not change the immobility time of control mice (Control + saline: n = 7; Control + sCT: n = 7). (D) In SPT, sCT also could not change the sucrose preference of control mice (Control + saline: n = 8; Control + sCT: n = 7). The data were analyzed by Unpaired t test. ns, no significance.
